# Do Computed Tomography Findings Affect Operating Time in Bi-Lateral Sagittal Split Osteotomy? A Pilot Study

**DOI:** 10.3390/diagnostics16091397

**Published:** 2026-05-06

**Authors:** Kazuyuki Yusa, Nobuyuki Sasahara, Tomoharu Hemmi, Satoshi Kasuya, Kenta Kagami, Kotaro Taniguchi, Shigeo Ishikawa

**Affiliations:** 1 Department of Dentistry, Oral and Maxillofacial-Plastic and Reconstructive Surgery, Faculty of Medicine, Yamagata University, Yamagata 990-9585, Japan; nsasahara@med.id.yamagata-u.ac.jp (N.S.); henmi@med.id.yamagata-u.ac.jp (T.H.); k-yonemura_s1117126@tky.ndu.ac.jp (K.K.); tanikota0125@gmail.com (K.T.); shigeo_ishikawa2011@yahoo.co.jp (S.I.); 2 Department of Dentistry, Oral and Maxillofacial Surgery, Okitama Public General Hospital, Yamagata 992-0601, Japan; silversoarer0315@gmail.com

**Keywords:** orthognathic surgery, sagittal split osteotomy, operating time

## Abstract

**Background/Objectives**: The aim of this study was to investigate the association between patient demographics and overall operating time during bilateral sagittal split osteotomy (BSSO). **Methods**: For this retrospective study, data were collected from patients who had undergone BSSO in our hospital between 2016 and 2023. The mandibular body and mandibular ramus were evaluated from preoperative computed tomography (CT), and CT attenuation values of cortical and cancellous bone in the mandibular ramus were obtained from standardized preoperative CT images. Patient demographics (age, sex, occlusal class, and body weight) before surgery were also collected from the medical record. **Results**: Forty-six patients were included in this study. Weight and CT attenuation of the mandibular ramus (both cortical and cancellous bone) correlated with operating time (weight: rs = 0.304, *p* = 0.04; CT attenuation of mandibular ramus: rs = 0.323, *p* = 0.029). In addition, the Mann–Whitney U test revealed significantly greater operating time in males (*p* < 0.05). Effects of each variable were estimated after adjusting for other variables, and CT attenuation of the mandibular ramus (both cortical and cancellous bone) (B = 0.088, *p* = 0.008) was identified as having an effect on operating time. Higher CT attenuation, reflecting greater cortical and cancellous bone density, may increase resistance during osteotomy and consequently prolong operating time. **Conclusions**: This pilot study observed a possible association between CT attenuation of the mandibular ramus and operating time in BSSO. However, these findings are preliminary and do not imply any causal relationships. Thus, further studies with larger cohorts are required to confirm these observations.

## 1. Introduction

Orthognathic surgery, including Le Fort I (LF1) osteotomy and bilateral sagittal split osteotomy (BSSO), is an effective procedure for correcting dentofacial deformities. BSSO is indicated for numerous cases, including mandibular prognathism, retrognathia, open bite, and facial asymmetry. Due to its versatility, BSSO is performed either as a standalone procedure or in combination with LF1 osteotomy in many cases. The primary goal of these surgeries is to improve occlusal function [1] and quality of life [2]. Ensuring safety is therefore of utmost importance in orthognathic surgery. For these reasons, extensive research has been conducted to date, including studies on the surgical precision [3] and complications [4] of orthognathic surgery. Among the procedures used in orthognathic surgery, BSSO is considered a technically demanding but fundamental operation. The procedure requires precise osteotomy of the mandibular ramus while carefully preserving the inferior alveolar nerve. Furthermore, anatomical variations in mandibular morphology may influence the technical difficulty of the procedure. These anatomical characteristics may also affect the time required for osteotomy, splitting, and fixation of the mandible during surgery. Consequently, patient-specific anatomical factors may contribute to variability in the operating time for BSSO.

From the perspective of ensuring surgical safety, clinical research has examined operating time across various fields, including gastrointestinal surgery, orthopedic surgery, and urology [5,6,7]. Further, numerous reports have indicated that prolonged operating time contributes to the occurrence of various complications [8,9,10]. By linking studies examining factors contributing to prolonged operating time with those investigating changes in complication rates or the occurrence of adverse events resulting from extended operating time, surgical safety can be maximized.

With respect to orthognathic surgery, many reports have discussed the operating time for orthognathic surgery, but most of those studies have treated operating time as an independent variable while primarily reporting effects prolonging operating time, such as increased incidence of complications or greater blood loss [11,12,13]. In contrast, studies specifically investigating the factors that influence operating time in orthognathic surgery remain limited. Preoperative imaging plays an essential role in the planning of orthognathic surgery. In particular, CT images allow surgeons to evaluate detailed anatomical structures of the mandible, including bone morphology, cortical thickness, and bone density. Such information may provide valuable insight into the potential technical difficulty of the surgical procedure. Previous studies have reported that bone morphology and cortical thickness can influence the surgical difficulty of orthognathic procedures. Aarabi M et al. reported that mandibular anatomic differences can increase the risk of a bad split during BSSO surgery [14]. Additionally, Kuroyanagi N et al. reported that the greater protrusion of the medial oblique ridge, thickened ramus, and longer distance from the mandibular incisors to the posterior border of the mandible may increase the surgical time and blood loss in patients undergoing BSSO [15]. However, quantitative analyses examining the relationship between CT-derived parameters and operating time during orthognathic surgery have rarely been reported.

Therefore, the aim of this study was to investigate the association between patient demographics, including various measurements obtained from preoperative CT, and the overall operating time during BSSO. We hypothesized that bone area and CT attenuation values extracted from CT images may be associated with operating time.

## 2. Materials and Methods

### 2.1. Patients

For this study, data were collected from patients who had undergone BSSO in the Department of Dentistry, Oral and Maxillofacial Surgery at Yamagata University Hospital between 2016 and 2023. Patients who underwent BSSO for the correction of mandibular prognathism, retrognathia, or facial asymmetry were included in this study. All patients had completed presurgical orthodontic treatment before surgery. Patients with craniofacial syndromes, history of mandibular trauma, or previous mandibular surgery were excluded to avoid potential confounding factors affecting mandibular morphology. Furthermore, patients with incomplete data, concomitant LF1 osteotomy, and/or genioplasty were excluded. All surgeries were performed by 5 experienced surgeons with at least 5 years of experience in orthognathic surgery at our hospital. While anatomical variations such as lingual cortical plate thickness, mandibular canal position, ramus width and curvature, and resistance during splitting may influence operative time and risk of unfavorable fractures, all surgeries were performed following standard protocols to minimize variability. Differences among surgeons and patient-specific anatomical variations are acknowledged as potential limitations of this study. All procedures performed in studies involving human participants were conducted in accordance with the ethical standards of the institutional and national research committees and the Declaration of Helsinki and its later amendments. Approval was obtained from the ethics committee at Yamagata University Faculty of Medicine (approval no. 2024-333; date of approval: 25 March 2025). This retrospective, observational study was conducted using the opt-out method of assuming consent via our hospital website.

### 2.2. Surgery and Data Analysis

**Figure 1:**
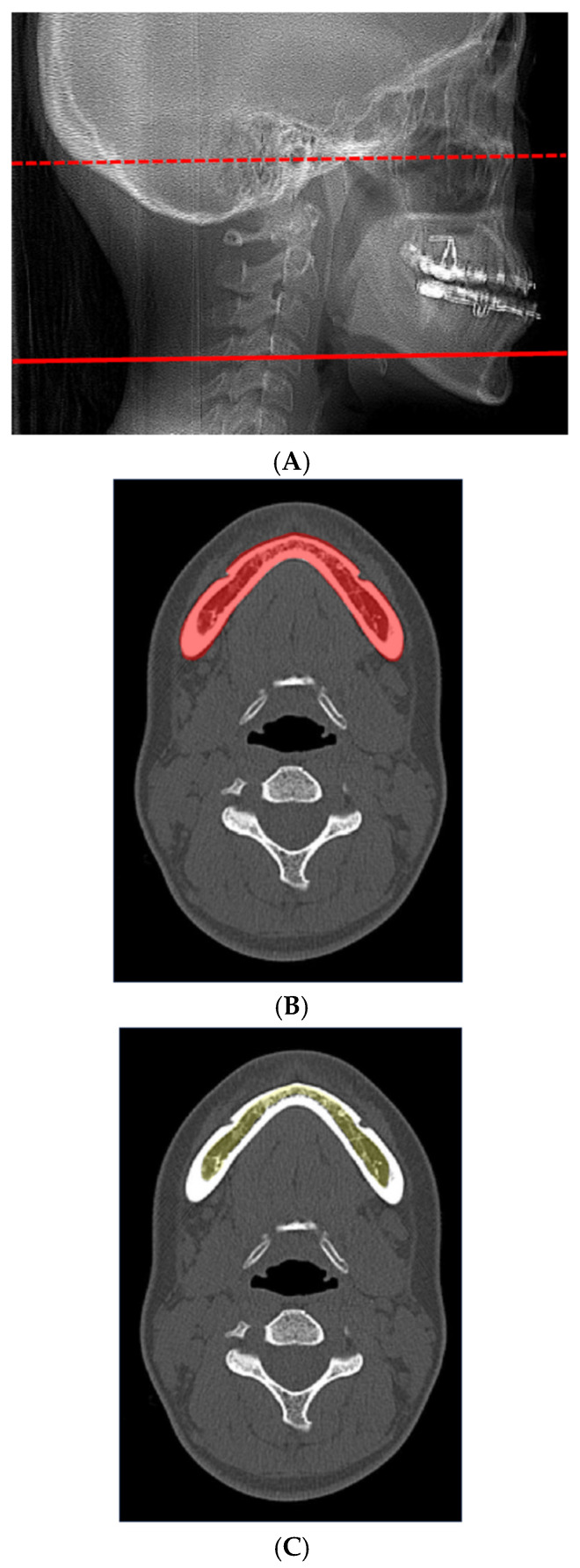
Mandibular body measurements. (**A**) Lateral CT for measurements of the mandibular body. FH plane, dotted line; measurement position, solid line. (**B**) Cortical and cancellous bone is extracted from axial-view CT of the mandibular body (red). (**C**) Cancellous bone is extracted from axial-view CT of the mandibular body (yellow). ROIs were placed in the mandibular body, including cortical (outer layer) and cancellous (trabecular) bone, avoiding soft tissues and teeth. A slice parallel to the FH plane, including the mental foramen, was used.

**Figure 2:**
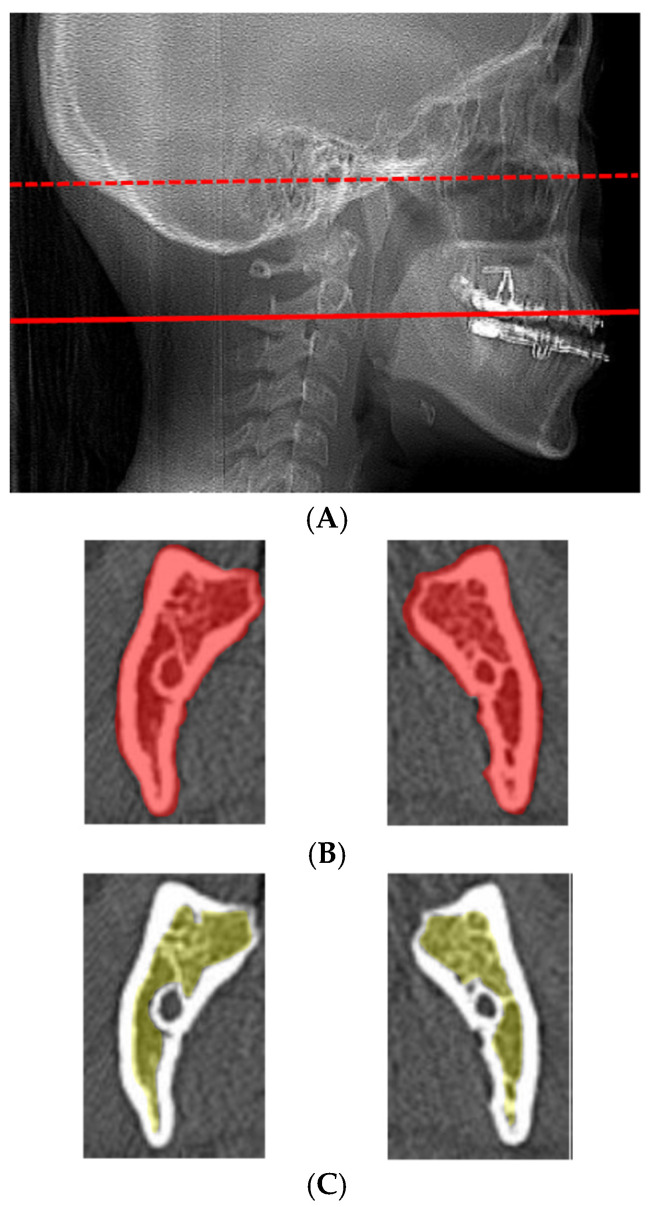
Mandibular ramus measurements. (**A**) Lateral CT for measurements of the mandibular body. FH plane, dotted line; measurement position, solid line. (**B**) Cortical and cancellous bone is extracted from axial-view CT of the mandibular ramus (red). (**C**) Cancellous bone is extracted from axial-view CT of the mandibular body (yellow). ROIs were placed in the mandibular ramus, including cortical and cancellous bone, avoiding soft tissues and teeth. A slice parallel to the FH plane through the second molar was used, and left and right sides were averaged.

BSSO was performed as modified by Dal Pont and Hunsuck. A horizontal bone groove and lateral cortical bone groove in the region between the first and second molars to the inferior border were made using a fissure bur. The two cortical bone grooves were connected with a piezoelectric device (VarioSurg 3; NSK-Nakanishi, Tochigi, Japan). The outer cortical bone and inner cancellous bone were then separated with a chisel and bone-separator forceps. Care was taken to avoid injury to the inferior alveolar nerve during osteotomy. The splitting procedure was performed carefully to prevent unfavorable fractures, commonly referred to as “bad splits,” which are known complications of BSSO. After mobilization of the distal segment, occlusion was confirmed using an intermediate surgical splint before rigid fixation was performed. Proximal and distal bone segments were then fixed using 4 or 6 monocortical screws. Preoperative CT images were obtained using multi-detector CT scanners with a slice thickness of 0.5 mm. Scan parameters were as follows: tube voltage was set at 120 kV, and tube current ranged from 40 to 100 mA depending on the scanner. For bone evaluation, both soft-tissue and bone reconstruction kernels were applied: soft-tissue conditions used FC03 AIDR 3D mild or J30s medium smooth SAFIRE 2, and bone conditions used FC30 AIDR 3D weak or H60s sharp FR SAFIRE(-). The field of view was 240 mm for all scans. These parameters were selected according to standard clinical protocols to ensure sufficient image quality for reliable measurement of cortical and cancellous bone. Regions of interest (ROIs) were manually defined within the mandibular body and ramus to measure CT attenuation values (Hounsfield units), reflecting bone density. ROIs were placed to include both cortical and cancellous bone while avoiding surrounding soft tissues and dental structures. All ROIs were manually placed based on consistent anatomical landmarks and radiological characteristics to ensure reproducibility. The size of each ROI was standardized relative to local mandibular dimensions to ensure consistency across patients. Care was taken to exclude surrounding soft tissues and dental structures when defining the ROIs. Higher HU values generally correspond to greater bone density and increased cortical bone thickness. For measurement of the mandibular body, a slice parallel to the Frankfort horizontal (FH) plane and including the mental foramen was selected on preoperative CT (Figure 1A–C). For measurements of the mandibular ramus, a slice parallel to the FH plane along the extension of a line passing through the second molar was selected for analysis (Figure 2A–C), and the average of left and right sides was calculated. Cortical bone was defined as the high-attenuation outer layer of the mandible, while cancellous bone was defined as the internal trabecular region with lower attenuation. To assess measurement reliability, all measurements were performed twice by the same examiner with a four-week interval, and the average value was used for the analysis. Operating time was defined as the duration from the initial surgical incision to completion of wound closure under general anesthesia. This definition was selected as a composite measure reflecting the overall surgical procedure, including non-bone-related steps (e.g., plating and wound closure). Although this measure includes steps not directly related to bone characteristics, it was used consistently and was a clinically relevant parameter across all cases. All operating times were obtained from the official surgical records maintained at our hospital. Information on patient demographics (age, sex, occlusal class, and body weight) before surgery was collected from the medical records.

### 2.3. Statistical Analysis

All statistical analyses were performed using Jeffreys’s Amazing Statistics Program (JASP 0.96; University of Amsterdam, Amsterdam, The Netherlands). Correlations between variables and operating time were assessed using the non-parametric Spearman’s rank correlation technique. The Mann–Whitney U test was used to examine the relationship between operating time and sex, as well as occlusal class. In addition, associations of sex, weight, and CT attenuation of the ramus were evaluated using multiple linear regression. Weight and CT attenuation of the ramus were treated as continuous variables, while sex was treated as a categorical variable. The level of significance was set as *p* < 0.05. ChatGPT (OpenAI, GPT-5.3) was used for statistical interpretation. The authors take full responsibility for the content.

## 3. Results

A total of 46 patients (31 women and 15 men; mean age, 25.8 ± 10.2 years) were included in this study. Occlusal class was 2 in seven cases and 3 in 39 cases. Mean weight was 58.1 ± 9.5 kg, and mean operating time was 130.8 ± 26.2 min (Table 1). Table 1 also shows cortical bone and cancellous bone, cancellous bone area, and CT attenuation for the mandibular body and mandibular ramus.

Table 2 shows correlations between variables (age, weight, and area and CT attenuation of the mandibular body and mandibular ramus) and operating time.

Among the examined variables, patient weight and CT attenuation values of the mandibular ramus (both cortical and cancellous bone) demonstrated significant positive correlations with operating time (weight: rs = 0.304, *p* = 0.04; CT attenuation of the mandibular ramus: rs = 0.323, *p* = 0.029). In particular, higher CT attenuation values of the ramus were associated with longer surgical duration, suggesting that increased bone density in this region may increase the technical difficulty of the osteotomy procedure.

**Figure 3:**
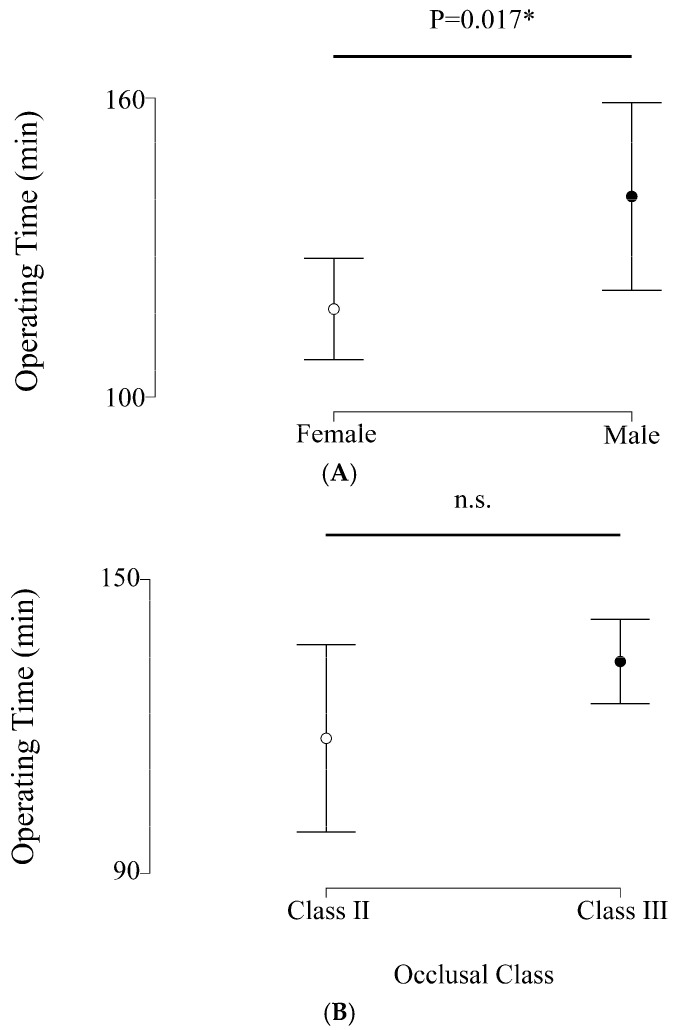
Operating times. (**A**) Operating times in female and male patients. (**B**) Operating times in occlusal class II and III. * *p*< 0.05.

In addition, the Mann–Whitney U test revealed significantly greater operating time in males (*p* < 0.05) (Figure 3A), but no significant difference in operating time was observed among the occlusal classes (Figure 3B). Further, Table 3 shows the results of the relationship between sex, weight, CT attenuation of mandibular ramus (both cortical and cancellous bone), and operating time using multiple linear regression.

Multiple linear regression analysis demonstrated that CT attenuation of the mandibular ramus (both cortical and cancellous bone) remained a significant predictor of operating time even after adjustment for sex and body weight (B = 0.088, *p* = 0.008). This finding indicates that bone characteristics of the ramus (both cortical and cancellous bone) may independently influence operating time.

## 4. Discussion

The present study aimed to identify factors associated with operating time for BSSO. BSSO is considered one of the most technically demanding procedures in orthognathic surgery. The osteotomy must be performed precisely while preserving the inferior alveolar nerve, and the splitting procedure requires careful control to avoid unfavorable fracture patterns.

Specifically, the effects of patient demographics, including age, sex, occlusal class, and weight, as well as bone area and CT attenuation values obtained from preoperative CT images, were examined in relation to the operating time required for BSSO. Understanding the factors that influence surgical duration is clinically important because prolonged operating time has been associated with an increased risk of intraoperative and postoperative complications in various surgical fields.

Regarding gender differences, male patients tended to show greater operating time in this study (Figure 3A). When discussing general surgery, men have been suggested to often experience longer operating times [16,17,18]. Ito et al. suggest that gender differences in retroperitoneal fat thickness are responsible for this phenomenon in retroperitoneal laparoscopic partial nephrectomy. Anatomical sex differences could conceivably influence surgical duration, depending on the type of procedure. On the other hand, some reports have found no difference in operating time between males and females for orthognathic surgery [19,20]. In the present study, although male patients tended to show longer operating times (Table 2), the results of the multiple linear regression analysis were not statistically significant (Table 3). This finding suggests that sex alone may not be a strong independent predictor of operative time for BSSO. Further studies involving larger cohorts will be necessary to clarify the influence of sex-related anatomical differences on surgical duration.

In this study, a similar trend was observed in the relationship between weight and operating time (Table 2 and Table 3). However, both weight and body mass index have been reported to correlate with operating time in various procedures [21,22]. One possible explanation is that increased soft-tissue thickness may reduce surgical visibility or make surgical manipulation more technically demanding. However, evidence regarding the relationship between body weight and operating time in orthognathic surgery remains limited. Because orthognathic procedures primarily involve bony structures rather than large soft-tissue dissection, the influence of body weight on surgical duration may be less pronounced compared with other surgical fields. Additional studies are therefore required to determine whether patient body habitus significantly affects operating time in BSSO.

In contrast, an exploratory finding of the present study revealed that the CT attenuation of the mandibular ramus was associated with operative time for BSSO, suggesting that bone density may be related surgical difficulty. CT attenuation values measured in Hounsfield units are widely used as indicators of bone density in various clinical fields, including implant dentistry and orthopedic surgery. Higher HU values are generally associated with increased cortical thickness and bone mineral density, which may increase resistance to osteotomy and splitting procedures, thereby contributing to greater surgical difficulty. In clinical practice, increased cortical bone thickness is often associated with greater difficulty in osteotomy. In addition, even after chisel-assisted splitting, segment separation may not proceed smoothly in cases with certain cancellous bone characteristics. CT attenuation values, reflecting both cortical and cancellous bone components, may partly represent these characteristics. CT attenuation values are generally considered to reflect bone density and bone quality. Higher CT attenuation values may be associated with greater force or more time during osteotomy and bone-splitting procedures. Conversely, lower CT attenuation values may indicate less dense bone structures that can be separated more easily during the splitting procedure. These differences in bone quality may therefore influence the technical difficulty of osteotomy and contribute to variations in operating time in BSSO.

Interestingly, no significant correlations were observed between operative time and the area or CT attenuation values of the mandibular body, the area of the mandibular ramus, or the area or CT attenuation of cancellous bone within the mandibular ramus. This finding suggests that bone quality specifically in the mandibular ramus may play a more important role in determining surgical difficulty than other mandibular regions. Because osteotomy and splitting during BSSO are primarily performed in the ramus region, the bone characteristics of this area may have a particularly strong influence on operative time.

In BSSO, management of the fracture line on the lingual side of the ramus is extremely important and often technically challenging. Bad splits in BSSO are often attributed to medial osteotomy, and the incidence according to surgical technique has been discussed [23,24]. Several reports have also addressed bone quality, suggesting that reduced trabecular bone thickness may represent a risk factor for bad splits [25,26]. Wang et al. reported that a short mandibular ramus is associated with anatomical features that increase the likelihood of a bad split [27]. Although no bad splits were observed during the study period in the present investigation, the finding that bone quality in the ramus region affects operative time is noteworthy and appears logically consistent with previous reports. Variations in bone density may influence the resistance encountered during osteotomy and splitting, thereby affecting the time required to safely complete the procedure.

Prolonged operating time is a critical factor in perioperative management, as it may lead to various complications [28]. Among these, intraoperative blood loss is one of the most troublesome complications, and numerous studies have evaluated this issue in relation to multiple factors [11,12,29]. Although the present study did not directly evaluate blood loss or postoperative complications, identifying factors that contribute to prolonged operating time may indirectly help reduce perioperative risks.

Another potential clinical implication of this study relates to surgical education and training. Because most orthognathic surgeries are elective procedures performed to improve quality of life, maintaining consistent surgical outcomes is essential regardless of the surgeon’s level of experience. Preoperative identification of cases that may require longer operative times could be helpful when assigning cases to residents serving as primary surgeons. In the present study, all procedures were performed by experienced surgeons to maintain a consistent level of surgical proficiency. However, Kretschmer et al. reported that residents tend to require longer operative times for orthognathic surgery [30]. Preoperative prediction of surgical difficulty and duration is thus considered highly important for resident training. Because most orthognathic surgeries are elective procedures performed to improve quality of life, maintaining consistent surgical outcomes is essential regardless of the surgeon’s level of experience. Preoperative identification of cases that may require longer operative times could be helpful when assigning cases to residents serving as primary surgeons. In the present study, all procedures were performed by experienced surgeons to maintain a consistent level of surgical proficiency. Preoperative prediction of surgical difficulty and duration is thus considered highly important for resident training. Furthermore, if CT-derived bone density parameters can be used to predict operative time or surgical difficulty, surgeons may be able to better plan operative schedules and anticipate technically challenging cases before surgery.

This study has several limitations. First, it was a retrospective, single-center study with a limited sample size. A prospective study with an appropriately calculated sample size will be required in the future. Second, only BSSO procedures were included. Due to the anatomical complexity of the maxilla, we have not yet identified CT characteristics associated with operating time in LF1 osteotomy. Furthermore, while bleeding during LF1 osteotomy appears to depend largely on the characteristics of the surrounding soft tissue or the blood vessels in the immediate vicinity, it is still necessary to carry out additional research in order to more thoroughly determine whether factors such as the quality or morphology of the bone itself might also contribute in some way, and to investigate whether any such potential contributions are consistently reflected in specific CT findings. Third, it remains unclear whether the findings of this study are associated with intraoperative or postoperative complications, such as blood loss or surgical site infection. Although prolonged operating time has been associated with increased risk of complications in previous studies, the present study did not directly evaluate intraoperative blood loss or postoperative adverse events. Therefore, the clinical impact of longer operative times in our cohort remains to be determined in future prospective studies. As noted above, the small sample size may also have influenced these results, and additional studies with larger cohorts are warranted. A further limitation is the involvement of multiple surgeons, which may have introduced some variability in operative time, and the absence of a formal statistical assessment of measurement reliability, such as intraclass correlation coefficient (ICC) analysis. Although all surgeons followed a standardized protocol, and measurements were repeated and averaged, these factors do not undermine the main findings of the study but should be acknowledged as methodological considerations. Furthermore, another limitation of this study is that operating time includes surgical steps not directly related to bone characteristics, such as plating and wound closure. Therefore, it does not exclusively reflect bone-specific procedural difficulty, and the findings should be interpreted as associations with overall surgical duration. Since BSSO involves surgical time spent on procedures unrelated to bone manipulation, such as incision, dissection, plating, and wound suturing, an important future direction is to investigate the relationship between bone manipulation time, as recorded in detailed surgical time logs, and CT findings. Despite the presence of these limitations, from a detailed clinical perspective, the ability to reliably predict operative difficulty by utilizing preoperative CT imaging has the potential to substantially enhance the overall process of surgical planning and to improve patient safety outcomes. By providing surgeons with advanced information regarding cases that are likely to require longer operative times, it becomes possible to proactively allocate the necessary resources, personnel, and preparation in a manner that optimizes surgical efficiency and reduces intraoperative risk. Consequently, this line of research is expected to make a highly significant and meaningful contribution toward addressing the multitude of challenges that are inherently associated with orthognathic surgery, and toward the successful realization of its intended clinical and surgical objectives, thereby ultimately serving as a crucial, impactful, and noteworthy contribution to the broader field of surgical practice and research.

## 5. Conclusions

In conclusion, this pilot study observed a possible association between CT attenuation of the mandibular ramus and operating time in BSSO. Because most orthognathic surgeries are elective procedures performed to improve quality of life, consistent outcomes are generally expected regardless of the surgeon’s level of experience (resident or experienced surgeon). However, these findings are preliminary and do not imply any causal relationships. Thus, further studies with larger cohorts are required to confirm these observations.

## Figures and Tables

**Table 1:** Characteristics of the 46 patients.

Age (Years) Mean ± SD Range		25.8 ± 10.2 16–59
Sex	Female; n (%)	31 (64.6)
	Male; n (%)	15 (31.3)
Occlusal class		
	II; n (%)	7 (14.6)
	III; n (%)	39 (81.3)
Weight (kg)		
	Mean ± SD	58.1 ± 9.5
Mandibular body		
Cortical and cancellous bone		
(mm^2^)	Mean ± SD	1116.3 ± 344.8
(HU)	Mean ± SD	905.8 ± 130.8
Cancellous bone		
(mm^2^)	Mean ± SD	530.6 ± 195.0
(HU)	Mean ± SD	321.0 ± 402.7
Mandibular ramus *		
Cortical and cancellous bone		
(mm^2^)	Mean ± SD	297.3 ± 54.5
(HU)	Mean ± SD	840.7 ± 110.0
Cancellous bone		
(mm^2^)	Mean ± SD	117.9 ± 60.2
(HU)	Mean ± SD	227.4 ± 101.8
Operating time (min)		
	Mean ± SD	130.8 ± 26.2

* Average of left and right measurements.

**Table 2:** Correlation between operating time and age, weight, and measurement values related to the mandible.

Variables	Rs	*p*
Age	−0.135	0.372
Weight	0.304	0.04 *
Mandibular body		
Cortical and cancellous bone		
(mm^2^)	0.101	0.503
(HU)	0.230	0.125
Cancellous bone		
(mm^2^)	−0.016	0.918
(HU)	0.158	0.293
Mandibular ramus		
Cortical and cancellous bone		
(mm^2^)	0.041	0.786
(HU)	0.323	0.029 *
Cancellous bone		
(mm^2^)	−0.180	0.230
(HU)	0.227	0.129

Statistically significant: * *p* < 0.05; rs, Spearman’s rank correlation coefficient.

**Table 3:** Multiple linear regression analysis of factors associated with operating time.

Variables	B	SE	β	*p*
Sex (M/F)	12.697	8.985		0.165
Weight	0.591	0.451	0.213	0.197
Mandibular ramus Cortical and cancellous bone (HU)	0.088	0.031	0.369	0.008 **

Statistically significant: ** *p* < 0.01. B, unstandardized regression coefficient; SE, Standard Error; β, Standardized regression coefficient.

## Data Availability

The data presented in this study are available upon request from the corresponding author due to privacy.

## Abbreviations

The following abbreviations are used in this manuscript:

BSSO	bilateral sagittal split osteotomy
CT	computed tomography
LF1	Le Fort I
FH	Frankfort horizontal

## References

[1] Moroi A., Ishihara Y., Sotobori M., Iguchi R., Kosaka A., Ikawa H., Yoshizawa K., Marukawa K., Ueki K. (2015). Changes in occlusal function after orthognathic surgery in mandibular prognathism with and without asymmetry. Int. J. Oral Maxillofac. Surg..

[2] Meger M.N., Fatturi A.L., Gerber J.T., Weiss S.G., Rocha J.S., Scariot R., Wambier L.M. (2021). Impact of orthognathic surgery on quality of life of patients with dentofacial deformity: A systematic review and meta-analysis. Br. J. Oral Maxillofac. Surg..

[3] Yi J.R., Yeweng S.J., Wu Z.X. (2023). Surgical Precision Analysis of Orthognathic Surgery Combined With Invisible Orthodontic. J. Craniofac Surg..

[4] Ferri J., Druelle C., Schlund M., Bricout N., Nicot R. (2019). Complications in orthognathic surgery: A retrospective study of 5025 cases. Int. Orthod..

[5] Abella M., Angeles J.P.M., Finlay A.K., Amanatullah D.F. (2023). Does Operative Time Modify Obesity-related Outcomes in THA?. Clin. Orthop. Relat. Res..

[6] Akman T., Binbay M., Akcay M., Tekinarslan E., Kezer C., Ozgor F., Seyrek M., Muslumanoglu A.Y. (2011). Variables that influence operative time during percutaneous nephrolithotomy: An analysis of 1897 cases. J. Endourol..

[7] Lowndes B., Thiels C.A., Habermann E.B., Bingener J., Hallbeck S., Yu D. (2016). Impact of patient factors on operative duration during laparoscopic cholecystectomy: Evaluation from the National Surgical Quality Improvement Program database. Am. J. Surg..

[8] Martin A.N., Tzeng C.D., Arvide E.M., Skibber J.M., Chang G.J., Nancy You Y.Q., Bednarski B.K., Uppal A., Dewhurst W.L., Cristo J.V. (2023). Impact of cumulative operative time on postoperative complication risk in simultaneous resections of colorectal liver metastases and primary tumors. HPB.

[9] de Angelis P., Tan K.S., Chudgar N.P., Dycoco J., Adusumilli P.S., Bains M.S., Bott M.J., Downey R.J., Huang J., Isbell J.M. (2023). Operative Time is Associated With Postoperative Complications After Pulmonary Lobectomy. Ann. Surg..

[10] Haeuser L., Marchese M., Noldus J., Kibel A.S., Carvalho F., Preston M.A., Cooper Z., Trinh Q.D., Mossanen M. (2023). Association between Operative Time and Short-Term Radical Cystectomy Complications. Urol. Int..

[11] Topan C., Demirbas A.E., Doğruel F., Ümit K.K., Yaşlı S.O., Soylu E., Canpolat D.G. (2025). Factors influencing intraoperative blood loss in bimaxillary orthognathic surgery. Med. Oral Patol. Oral Cir. Bucal.

[12] Thastum M., Andersen K., Rude K., Nørholt S.E., Blomlöf J. (2016). Factors influencing intraoperative blood loss in orthognathic surgery. Int. J. Oral Maxillofac. Surg..

[13] Yu C.N., Chow T.K., Kwan A.S., Wong S.L., Fung S.C. (2000). Intra-operative blood loss and operating time in orthognathic surgery using induced hypotensive general anaesthesia: Prospective study. Hong Kong Med. J..

[14] Aarabi M., Tabrizi R., Hekmat M., Shahidi S., Puzesh A. (2014). Relationship between mandibular anatomy and the occurrence of a bad split upon sagittal split osteotomy. J. Oral Maxillofac. Surg..

[15] Kuroyanagi N., Miyachi H., Kanazawa T., Kamiya N., Nagao T., Shimozato K. (2013). Morphologic features of the mandibular ramus associated with increased surgical time and blood loss in sagittal split-ramus osteotomy. J. Oral Maxillofac. Surg..

[16] Rudisill S.S., Eberlin C.T., Kucharik M.P., Linker J.A., Naessig S.A., Best M.J., Martin S.D. (2022). Sex differences in utilization and perioperative outcomes of arthroscopic rotator cuff repair. JSES Int..

[17] Bazoua G., Tilston M.P. (2014). Male gender impact on the outcome of laparoscopic cholecystectomy. JSLS J. Soc. Laparosc. Robot. Surg..

[18] Ito H., Makiyama K., Kawahara T., Osaka K., Izumi K., Yokomizo Y., Nakaigawa N., Yao M. (2016). The impact of gender difference on operative time in laparoscopic partial nephrectomy for T1 renal tumor and the utility of retroperitoneal fat thickness as a predictor of operative time. BMC Cancer.

[19] Olsen J.J., Ingerslev J., Thorn J.J., Pinholt E.M., Gram J.B., Sidelmann J.J. (2016). Can Preoperative Sex-Related Differences in Hemostatic Parameters Predict Bleeding in Orthognathic Surgery?. J. Oral Maxillofac. Surg..

[20] Rummasak D., Apipan B., Kaewpradup P. (2011). Factors that determine intraoperative blood loss in bimaxillary osteotomies and the need for preoperative blood preparation. J. Oral Maxillofac. Surg..

[21] Lim S., Lee S.S., Oh J., Lee D.H. (2023). Weight Is a Predictor of Delayed Operation Time in Primary Isolated Anterior Cruciate Ligament Reconstruction. Biomedicines.

[22] Saiganesh H., Stein D.E., Poggio J.L. (2015). Body mass index predicts operative time in elective colorectal procedures. J. Surg. Res..

[23] Zeynalzadeh F., Shooshtari Z., Eshghpour M., Hoseini Zarch S.H., Tohidi E., Samieirad S. (2021). Dal Pont vs Hunsuck: Which Technique Can Lead to a Lower Incidence of Bad Split during Bilateral Sagittal Split Osteotomy? A Triple-blind Randomized Clinical Trial. World J. Plast. Surg..

[24] Arakeri G., Brennan P.A. (2015). A guiding oblique osteotomy cut to prevent bad split in sagittal split ramus osteotomy: A technical note. Plast. Aesthetic Res..

[25] Muto T., Shigeo K., Yamamoto K., Kawakami J. (2003). Computed tomography morphology of the mandibular ramus in prognathism: Effect on the medial osteotomy of the sagittal split ramus osteotomy. J. Oral Maxillofac. Surg..

[26] Telha W., Abotaleb B., Zhang J., Bi R., Zhu S., Jiang N. (2023). Correlation between mandibular anatomy and bad split occurrence during bilateral sagittal split osteotomy: A three-dimensional study. Clin. Oral Investig..

[27] Wang T., Han J.J., Oh H.K., Park H.J., Jung S., Park Y.J., Kook M.S. (2016). Evaluation of Mandibular Anatomy Associated With Bad Splits in Sagittal Split Ramus Osteotomy of Mandible. J. Craniofac Surg..

[28] Damrongsirirat N., Kaboosaya B., Siriwatana K., Subbalekha K., Jansisyanont P., Pimkhaokham A. (2022). Complications related to orthognathic surgery: A 10-year experience in oral and maxillofacial training center. J. Cranio-Maxillofac. Surg..

[29] Yusa K., Ishikawa S., Takagi A., Kunii S., Iino M. (2022). Bone marrow space volume of the mandible influencing intraoperative blood loss in bilateral sagittal split osteotomy: A pilot Study. J. Stomatol. Oral Maxillofac. Surg..

[30] Kretschmer W., Köster U., Dietz K., Zoder W., Wangerin K. (2008). Factors for intraoperative blood loss in bimaxillary osteotomies. J. Oral Maxillofac. Surg..

